# Indocyanine Green to Assess Vascularity of Ileal Conduit Anastomosis During Pelvic Exenteration for Recurrent/Persistent Gynecological Cancer: A Pilot Study

**DOI:** 10.3389/fonc.2021.727725

**Published:** 2021-12-07

**Authors:** Nicolò Bizzarri, Nazario Foschi, Matteo Loverro, Lucia Tortorella, Francesco Santullo, Andrea Rosati, Salvatore Gueli Alletti, Barbara Costantini, Valerio Gallotta, Gabriella Ferrandina, Anna Fagotti, Francesco Fanfani, Alfredo Ercoli, Vito Chiantera, Giovanni Scambia, Giuseppe Vizzielli

**Affiliations:** ^1^ Unità Operativa Complessa (UOC) Ginecologia Oncologica, Dipartimento per la salute della Donna e del Bambino e della Salute Pubblica, Fondazione Policlinico Universitario A. Gemelli, Istituto di Ricovero e Cura a Carattere Scientifico (IRCCS), Rome, Italy; ^2^ UOC Clinica Urologica, Dipartimento Scienze Mediche e Chirurgiche, Fondazione Policlinico Universitario A. Gemelli, IRCCS, Rome, Italy; ^3^ UOC Chirurgia Peritoneo e Retroperitoneo, Dipartimento Scienze Mediche e Chirurgiche, Fondazione Policlinico Universitario A. Gemelli, Istituto di Ricovero e Cura a Carattere Scientifico (IRCCS), Rome, Italy; ^4^ Università Cattolica del Sacro Cuore, Rome, Italy; ^5^ Department of Gynecologic Oncology and Minimally-Invasive Gynecologic Surgery, Università Degli Studi di Messina, Policlinico G. Martino, Messina, Italy; ^6^ ARNAS Ospedali Civico Di Cristina Benfratelli, Department of Gynecologic Oncology, University of Palermo, Palermo, Italy; ^7^ Obstetrics and Gynecology Department, Academic Hospital of Udine, Department of Medicine, University of Udine, Udine, Italy

**Keywords:** indocyanine green (ICG), pelvic exenteration (PE), ileal conduit diversion, major postoperative complications, anastomosis, gynecological cancer

## Abstract

**Introduction:**

Pelvic exenteration performed for recurrent/persistent gynecological malignancies has been associated with urological short- and long-term morbidity due to altered vascularization of tissues for previous radiotherapy. The aims of the present study were to describe the use of intravenous indocyanine green (ICG) to assess vascularity of urinary diversion (UD) after pelvic exenteration for gynecologic cancers, to evaluate the feasibility and safety of this technique, and to assess the postoperative complications.

**Methods:**

Prospective, observational, single-center, pilot study including consecutive patients undergoing anterior or total pelvic exenteration due to persistent/recurrent gynecologic cancers between August 2020 and March 2021 at Fondazione Policlinico Gemelli IRCCS, Rome, Italy. All patients underwent intravenous injection of 3–6 ml of ICG (1.25 mg/ml) once the UD was completed. A near-infrared camera was used to evaluate ICG perfusion of anastomoses (ileum–ileum, right and left ureter with small bowel, and colostomy or colorectal sides of anastomosis) a few seconds after ICG injection.

**Results:**

Fifteen patients were included in the study. No patient reported adverse reactions to ICG injection. Only 3/15 patients (20.0%) had an optimal ICG perfusion in all anastomoses. The remaining 12 (80.0%) patients had at least one ICG deficit; the most common ICG deficit was on the left ureter: 3 (20.0%) vs. 1 (6.7%) patient had no ICG perfusion on the left vs. right ureter, respectively (p = 0.598). 8/15 (53.3%) and 6/15 (40.0%) patients experienced grade ≥3 30-day early and late postoperative complications, respectively. Of these, two patients had early and one had late postoperative complications directly related to poor perfusion of anastomosis (UD leak, ileum–ileum leak, and benign ureteric stricture); all these cases had a suboptimal intraoperative ICG perfusion.

**Conclusion:**

The use of ICG to intraoperatively assess the anastomosis perfusion at time of pelvic exenteration for gynecologic malignancy is a feasible and safe technique. The different vascularization of anastomotic stumps may be related to anatomical sites and to previous radiation treatment. This approach could be in support of selecting patients at higher risk of complications who may need personalized follow-up.

## Introduction

Pelvic exenteration is a major radical operation that represents the last curative option in recurrent/persistent gynecologic cancers previously treated with radiotherapy (1, 2). In selected patients in whom this surgical procedure is performed with curative intent, overall survival ranges from 20% to 73% at 5 years (2–4). In view of the promising oncological outcomes, gynecologic oncologists have recently pushed the boundaries and stretched the indication of such surgery to patients with pelvic sidewall involvement (5, 6), previously deemed inoperable.

Different studies have reported the perioperative morbidity associated with pelvic exenteration (7). In particular, postoperative complications range from 51% to 82%, of which 22%–32% are major complications (1, 7). The anterior part of pelvic exenteration contemplates the reconstruction by urinary diversion (UD) that can be performed with different techniques (8). UD options (orthotopic neobladder, continent and incontinent diversions) require anastomosis of the ureters with selected bowel segments; among UD, ileal conduit continues to be the most widely UD used after pelvic exenteration for gynecologic malignancies (8–10). A wide range of complications are not only related to the pelvic exenteration but specifically to UD: 45% of patients experience functional complications after 5 years, 50% at 10 years, and 94% at 15 years (11). Despite the multiple variations in uretero-enteric anastomosis, ureteric fistula and benign ureteric stricture represent short- and long-term complications associated with ileal conduit urinary diversion in 5%–22% and 3%–27% of cases, respectively (8, 12).

Benign ureteric strictures are mainly related to ischemic-inflammatory mechanisms or surgical technique (poor preservation of ureteral vascular supply, ureteral handling and mobilization, tension of anastomosis, tightness of suture, etc.) (13, 14), and they are associated with urinary tract infections and potential detrimental effects on renal function.

Ischemia has been considered a key factor associated with anastomosis complications, leading to prolonged hospitalization, septic complications, hospital readmission, need for invasive interventions, and finally loss of the renal unit (15, 16).

Very recently, indocyanine green (ICG) fluorescence has been proposed as a tool to assess tissue perfusion. ICG becomes fluorescent when excited with near-infrared light, allowing for its detection within tissue with specially designed cameras. ICG has gained wide applicability in different fields of surgery, including the assessment of anastomosis and flap perfusion (17, 18). In particular, intravenously administered ICG is used to identify vessel perfusion and differentiate tissue density (19). Very few studies have assessed the use of intravenous ICG to check vascularity of ileal conduit UD, showing that ICG was useful to reduce the incidence of benign ureteric strictures (20, 21). However, no study was performed in patients with gynecologic cancers, particularly after treatment with pelvic radiotherapy.

The aims of the present study were to describe the use of ICG to assess vascularity of UD after pelvic exenteration for gynecologic cancers to evaluate the feasibility and safety of this technique and to assess the postoperative complications.

## Methods

**Figure 1:**
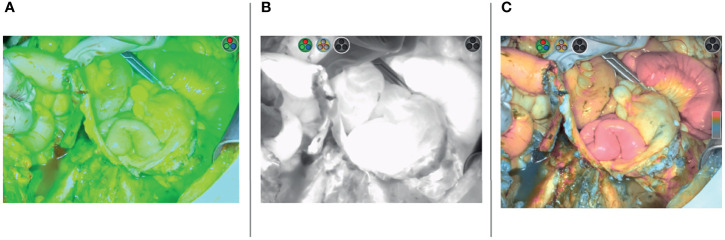
Wallace ileal conduit uretero-enteric anastomoses indocyanine green (ICG) perfusion demonstrated with three different modalities: overlay fluorescence mode **(A)**, contrast fluorescence mode **(B)**, color segmented fluorescence mode **(C)**.

This is a prospective, observational, pilot study. The present study was approved by the institutional review board (number DIPUSVSP-27-07-20108). Consecutive patients undergoing anterior or total pelvic exenteration due to persistent or recurrent gynecologic cancers between August 2020 and March 2021 at Fondazione Policlinico Gemelli IRCCS, Rome, Italy, were included. Women older than 18 years with histological diagnosis of cervical, vulvar, vaginal, or endometrial cancer previously treated with (chemo)radiotherapy or with chemotherapy only were included; patients with allergy to iodine, who underwent posterior exenteration only, and who underwent ureterostomy were excluded. Pelvic exenteration was performed with the attempt to remove pelvic organs in one single specimen and with the aim of achieving tumor-free surgical margins. The surgical approach was open or minimally invasive according to the patient’s body mass index (BMI) and to the tumor’s maximum diameter at preoperative imaging (22). All patients underwent intravenous injection of 3–6 ml of ICG (1.25 mg/ml) once the UD was completed. All ileal conduits were performed by the same urologist (NF), and the decision to perform Bricker–Nelaton vs. Wallace type I (medial-posterior wall continuous suture) (8) uretero-enteric anastomosis technique was taken according to the appearance, availability, and mobility of the distal ureters after the oncological resection and previous radiotherapy. A near-infrared laparoscopic camera (Olympus, Tokyo, Japan) or the SPY Portable Handheld Imager (SPY-PHI) (Stryker, Kalamazoo, MI, USA) was used to evaluate ICG perfusion a few seconds after intravenous ICG injection (**Figure 1A**). Contrast fluorescence mode demonstrated ICG fluorescence in perfused tissues as white against a black/gray background (**Figure 1B**). Color-segmented fluorescence was used to show different levels of ICG uptake demonstrating higher levels of perfusion (**Figure 1C**). After ICG injection, a four-tier (+++ vs. ++- vs. + - - vs. - - -) classification was used to assess the vascularity (ICG perfusion) of each anastomosis: ileum–ileum, right and left ureter with small bowel, and colostomy or colorectal sides of anastomosis (in case of total pelvic exenteration). Ureter perfusion was analyzed separately even in case of Wallace type I anastomosis. The classification of ICG perfusion of anastomoses was performed by two independent assessors: the urologist and the gynecologic oncologist. In case of conflict, a third opinion from a gynecologic oncologist was requested to solve the conflict. In view of the observational nature of the present study, no surgical action was taken in case of poor ICG perfusion of the anastomoses.

Postoperative complications were graded according to Clavien–Dindo classification system (23), and they were divided into early (between date of pelvic exenteration and 30th postoperative day) and late (from 31st to 180th postoperative day).

All patients underwent radiologic assessment of the abdomen 3 months after the pelvic exenteration (CT scan, MRI scan, or ultrasound scan) to check for any sign of hydronephrosis.

### Statistical Analysis

A sample of at least 12 patients was considered adequate for a descriptive pilot study (24).

**Figure 2:**
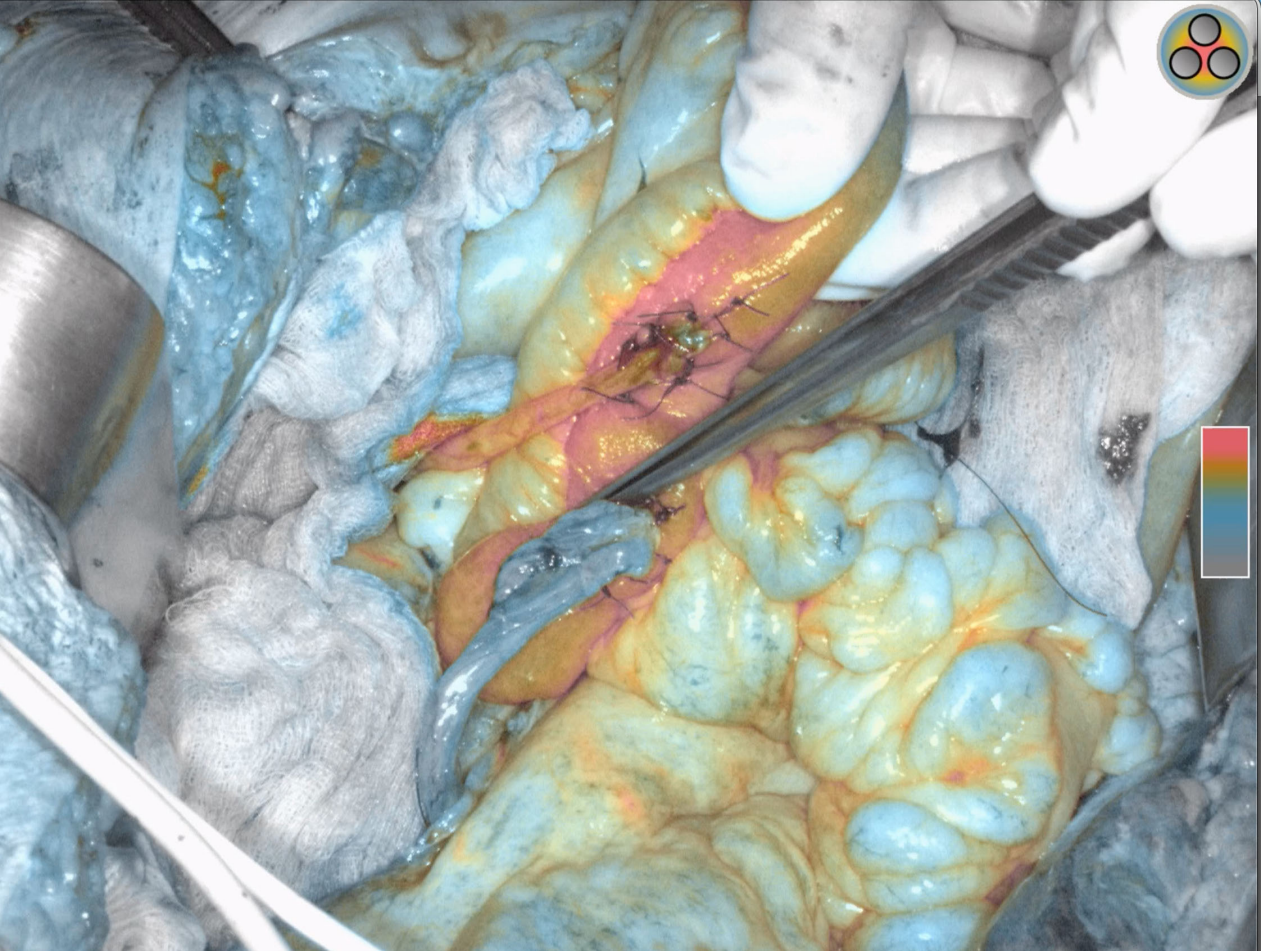
Bricker ileal conduit uretero-enteric anastomoses demonstrating right ureter with optimal indocyanine green (ICG) perfusion (+++) vs. left ureter with poor ICG perfusion (- - -).

Standard descriptive statistics was used: median with range and incidence with percentage were used to report continuous and categorical variables, respectively. Student’s t-test and chi-square test were used to compare continuous and categorical variables, respectively. ICG perfusion was assessed in two ways: comparing poor (- - -/+ - -) vs. good (++-/+++) (**Figure 2**) ICG perfusion and comparing any ICG deficit vs. optimal ICG vascularization (optimal vascularization was defined as such, if all anastomoses had maximum ICG perfusion +++). Logistic regression analysis was performed to estimate variables related to negative perioperative outcomes.

## Results

Fifteen patients were included in the study. Clinical characteristics of the entire cohort are demonstrated in **Table 1**. All but one patient (93.3%) underwent previous treatment with radiotherapy or chemoradiotherapy. Seven (46.7%) patients were operated on for persistent disease and eight (53.3%) for recurrent disease. The most common site of primary cancer was the cervix (12, 80.0%), and most patients underwent total pelvic exenteration (10, 66.7%). One (6.7%) patient underwent minimally invasive pelvic exenteration, while the remaining underwent open approach.

**Table 1:** Patients’ characteristics.

Characteristic	Patients (N = 15) (range, %)
**Age, median**	53 (32-74)
**BMI, median**	23.4 (17.0-35.2)
**Tumor origin**	
Cervix	12 (80.0)
Vulva	1 (6.7)
Endometrium	2 (13.3)
**ASA score**	
1	0
2	14 (93.3)
3	1 (6.7)
4	0
**Previous cancer treatment**	
Radio(chemotherapy)	14 (93.3)
Chemotherapy	1 (6.7)
**Type of pelvic exenteration**	
Anterior	5 (33.3)
Total	10 (66.7)
**Smoke**	
No	14 (93.3)
Yes	1 (6.7)
**Hyperthension**	
No	13 (86.7)
Yes	2 (13.3)
**Diabetes**	
No	13 (86.7)
Yes	2 (13.3)
**Months from last RT, median**	8 (1-21)
**30-day postoperative complications (Clavien-Dindo)**	
No	2 (13.3)
G1-2	5 (33.3)
G3-5	8 (53.3)
**Late postoperative complications (Clavien-Dindo)**	
No	6 (40.0)
G1-2	3 (20.0)
G3-5	6 (40.0)
**Type of ileal conduit**	
Bricker	9 (60.0)
Wallace type 1	6 (40.0)
**Dose of ICG injected**	
3 ml	14 (93.3)
6 ml	1 (6.7)

No patient reported adverse reactions to ICG injection. Only 3/15 patients (20.0%) had an optimal ICG perfusion (+++) in all anastomoses. The remaining 12 (80.0%) patients had at least one ICG deficit; the most common ICG deficit was on the left ureter: 3 (20.0%) vs. 1 (6.7%) patient had no ICG perfusion (- - -) on the left vs. right ureter, respectively (p = 0.598). Out of 30 ureters analyzed separately, 20 (66.7%) had good (++-/+++) while 10 (33.3%) had poor (- - -/+ - -) ICG perfusion. **Table 2** shows the grading of ICG perfusion in different anastomoses. No difference was seen when comparing poor (- - -/+ - -) vs. good (++-/+++) ICG perfusion between right vs. left ureter (p = 0.700). Moreover, both types of cameras were used and no differences were noted between them (data not shown).

**Table 2:** Indocyanine green (ICG) perfusion according to different anastomoses.

ICG perfusion of anastomosis/ostomy	Patients (N = 15) (%)
**Right ureter anastomosis**	
+ + +/+ + -	9 (60.0)
+ - -/- - -	6 (40.0)
**Left ureter anastomosis**	
+ + +/+ +-	11 (73.3)
+ - -/- --	4 (26.6)
**Ileum-ileum anastomosis**	
+ + +/+ + -	15 (100.0)
+ --/---	0
Colo-rectal anastomosis*	
+ + +/+ + -	3 (100.0)
+ - -/- - -	0
End colostomy**	
+ + +/+ + -	5 (71.4)
+ - -/- - -	2 (28.6)

*3 patients underwent total pelvic exenteration with colo-rectal anastomosis.

**7 patient underwent total pelvic exenteration with end sigmoid colostomy.

Two (13.3%) patients did not experience early postoperative complications. Among the remaining patients, five (33.3%) had grade 2, three (20.0%) had grade 3a, three (20.0%) had grade 3b, and two (13.3%) had grade 4 early postoperative complications. Two (13.3%) patients had early postoperative complications that can be directly attributed to an impaired vascularization of the anastomosis. One patient had a leak of uretero-enteric anastomosis in the Bricker ileal conduit: this patient had an intraoperative perfusion of the left ureter that was classified as - - -; the second patient had a leak of ileo-ileal anastomosis: this anastomosis was intraoperatively classified as ++-. No patient developed necrotic ischemia of the ileal conduit.

The median time of follow-up was 4.5 months (range, 1–7). Six (40.0%) patients did not experience late postoperative complications; three (20.0%) patients had grade 2, three (20.0%) patients had grade 3a, and one (6.7%) patient had grade 3b. Two patients (13.3%) died of late complications: one had a fistula between external iliac artery and small bowel with following sepsis, and the other had a sepsis of pulmonary origin. Regarding anastomosis vascularity complications, one patient (6.7%) had left benign ureteric stricture at 47th postoperative day. The left ureter in this patient was intraoperatively judged with a - - - ICG perfusion.

**Table 3** demonstrates the correlation between ICG perfusion of all anastomoses and clinical outcomes. None of the analyzed outcomes was significantly related to a poor or a better ICG perfusion of all anastomoses.

**Table 3:** Correlation between indocyanine green (ICG) perfusion of all anastomoses* and clinical outcomes.

Clinical outcome	Poor vascularization (---/+ --/+ + -) (N = 12)	Optimal vascularization (+ + +) (N = 3)	p-value
**Early complications**			0.448
No	2 (16.7)	0	
Yes	10 (83.3)	3 (100.0)	
**Early complications**			0.569
No/G1-2	5 (41.7)	2 (66.7)	
G3-5	7 (58.3)	1 (33.3)	
**Late complications**			0.792
No	5 (41.7)	1 (33.3)	
Yes	7 (58.3)	2 (66.7)	
**Late complications**			0.812
Not yet beyond 30 days	1 (8.3)	0	
No/G1-2	6 (50.0)	2 (66.7)	
G3-5	5 (41.7)	1 (33.3)	
**Postoperative urinary fistula**	1 (8.3)	0	0.605
**Hydronephrosis at 3 months**	5 (41.7)	0	0.287

*Optimal vascularization was defined as such, if all anastomoses had maximum ICG perfusion (+ + +).

When we compared the type of uretero-enteric anastomosis technique in UD performed, we noted that patients who underwent Bricker anastomosis technique had a higher chance to have any deficit in ICG perfusion compared to Wallace type I anastomosis technique (9, 100.0% in Bricker vs. 3, 50.0% in Wallace type I; p = 0.044). When we analyzed the 30 ureters separately, we noted that there was no difference in the ICG perfusion between the two anastomosis techniques (good perfusion ++-/+++ in 10/18, 55.6% vs. 10/12, 83.3% patients, respectively; p = 0.235).

Similarly, there was no statistically significant relation between the type of early postoperative complication (no complications/G1–2 vs. G3–4) and the ICG perfusion to any anastomosis (p = 0.569). Moreover, no difference between no/G1–2 vs. G3–4 late complications according to ICG perfusion was noted (p = 0.812) (**Table 3**).

**Table 4** represents a logistic regression analysis of variables associated with 30-day grade 3–4 postoperative complications. None of the analyzed variables was significantly associated with early postoperative complications.

**Table 4:** Logistic regression analysis of variables associated with 30-day grade 3–4 postoperative complications.

Characteristic	OR (95%CI)	p-value
**Age**	0.500 (0.35-7.104)	0.609
<65		
≥65		
**ICG perfusion of anastomoses**	2.800 (0.196-40.057)	0.448
Poor		
Optimal*		
**Hypertension**	0.857 (0.044-16.851)	0.919
No		
Yes		
**Diabetes**	0.857 (0.044-16.851)	0.919
No		
Yes		
**Months from last radiotherapy**	5.000 (0.388-63.387)	0.217
<6		
≥6		
**Type of Pelvic Exenteration**	1.500 (0.170-13.225)	0.715
Anterior		
Total		
**Type of ileal conduit**	2.500 (0.292-21.399)	0.403
Bricker		
Wallace		

*Optimal vascularization was defined as such, if all anastomoses had maximum ICG perfusion (+ + +).

## Discussion

With the present study, we aimed to describe the use of ICG to assess vascularity of ileal conduit UD, uretero-enteric anastomosis, and intestinal anastomosis after pelvic exenteration for gynecologic cancers and to assess postoperative complications. With the power of a pilot study, therefore with a limited number of patients, we showed that only 20.0% of patients had optimal ICG perfusion in all anastomoses; this is most likely due to the fact that almost all included patients (93.3%) underwent previous treatment with radio(chemo)therapy. To the best of our knowledge, this is the first report to describe the use of ICG to assess vascularity of UD, uretero-enteric anastomosis, and ileo-ileal anastomosis in patients who previously underwent radiotherapy; moreover, it is the first study that assess ileal conduit perfusion with ICG in patients with gynecologic malignancies.

The main objective of intraoperative assessment of anastomosis vascularization is to reduce the incidence of postoperative complications that may be directly related to poor perfusion, such as leak/fistula and benign strictures. In our series, we documented one leak of uretero-enteric anastomosis and one benign ureteric stricture; in both cases, there was a poor vascularization of the ureteric stump (- - -). Interestingly, this happened in the left ureter; even though not statistically significant, the left ureter was more frequently impacted by a poor vascularization. This may be due to the mobilization of the left ureter during the formation of the ileal conduit with consequent perfusion impairment (15). Moreover, when looking at the vascularization of patients with Bricker vs. Wallace type I uretero-enteric anastomosis, we noted that Bricker conduit was less likely to have both ureters with optimal vascularization; nevertheless, there is large evidence in literature showing no difference in short- and long-term outcomes when Bricker is compared to Wallace techniques (25).

Few urology studies have reported the use of ICG to intraoperatively assess vascularization of uretero-enteric anastomosis after radical cystectomy (13, 14). The main result reported by these Authors was the reduced incidence of benign ureteric strictures when perfusion was analyzed with ICG. Particularly, poor distal perfusion required further ureter re-excision, leading to a significant per-ureter reduction of ureteric strictures [from 7.5% to 0% in one study (13) and from 6.6% to 0% in the other study (14)]. The present study was designed as a prospective observational study to assess the feasibility and safety of ICG use after radiation therapy in patients undergoing pelvic exenteration and to evaluate the outcomes of different ICG perfusions; therefore, the surgery was not modified according to intraoperative findings. Further interventional studies with re-excision of poorly perfused stumps may be necessary to better understand the potential role of ICG to reduce short- and long-term postoperative complications.

The main limitations of the present study are represented by the small sample size and the short follow-up period that prevent to draw solid conclusions. Selection bias to choose UD anastomosis technique may also be considered as a potential limitation such as the subjectivity of the evaluation. However, since the introduction of artificial intelligence is revolutionizing surgery, the combination of endoscopy with fluorescence imaging with ICG read by bioinformatics software could be in the future a forward-looking modality to enhance surgical precision and reduce postoperative complications while improving oncologic and functional outcomes with a better quality of life.

Other potential biases of the perfusion of the ileal conduit such as BMI or concomitant cardiovascular diseases were not investigated in this paper due to its small sample size. Nevertheless, this is the first report on the use of ICG to assess ileal conduit perfusion in the setting of pelvic exenteration in gynecologic malignancy after pelvic radiotherapy and reports a graduated scale of stump perfusion.

## Conclusion

The use of ICG to intraoperatively assess anastomosis perfusion at the time of pelvic exenteration for gynecologic malignancy is a feasible and safe technique. The different vascularization of anastomotic stumps may be related to anatomical sites and to previous radiation treatment. This approach could be in support of selecting patients at higher risk of complications who may need personalized follow-up. In this context, further studies are required to better understand whether ICG may be able to improve morbidity outcomes in this setting.

## Data Availability Statement

The original contributions presented in the study are included in the article/supplementary material. Further inquiries can be directed to the corresponding author.

## Ethics Statement

The studies involving human participants were reviewed and approved by the institutional review board of Fondazione Policlinico Gemelli IRCCS (number DIPUSVSP-27-07-20108). The patients/participants provided their written informed consent to participate in this study.

## Author Contributions

All authors fulfilled the conditions required for authorship and approved the submission, in particular: GV and NB provided a substantial contribution to the design of the project and drafted the article. NF, ML, LT, FS, AR, SGA BC, and VG collected the data giving an important contribution to critical aspects of the research. GF, AF, and FF revised the paper for important intellectual content. GS approved the final version to be published. All authors contributed to the article and approved the submitted version.

## Conflict of Interest

The authors declare that the research was conducted in the absence of any commercial or financial relationships that could be construed as a potential conflict of interest.

The handling editor and reviewers AG and GC declared a shared affiliation with several of the authors, NB, NF, ML, FS, AR, SG, BC, VG, GF, AF, FF, GS, and GV, at time of review.

## Publisher’s Note

All claims expressed in this article are solely those of the authors and do not necessarily represent those of their affiliated organizations, or those of the publisher, the editors and the reviewers. Any product that may be evaluated in this article, or claim that may be made by its manufacturer, is not guaranteed or endorsed by the publisher.
